# Lay HIV counselors' knowledge and attitudes toward depression: A mixed-methods cross-sectional study at primary healthcare centers in Mozambique

**DOI:** 10.3389/fpubh.2022.919827

**Published:** 2022-09-28

**Authors:** Flavio Mandlate, M. Claire Greene, Luis F. Pereira, Annika C. Sweetland, Donald Kokonya, Cristiane S. Duarte, Francine Cournos, Maria A. Oquendo, Milton L. Wainberg, Mohsin Sidat, Esperança Sevene, Marcelo F. Mello

**Affiliations:** ^1^Department of Mental Health, Ministry of Health, Maputo, Mozambique; ^2^Department of Psychiatry, Federal University of Sao Paulo (UNIFESP), Sao Paulo, Brazil; ^3^Program on Forced Migration and Health, Heilbrunn Department of Population and Family Health, Columbia University Mailman School of Public Health, New York, NY, United States; ^4^New York State Psychiatric Institute, Columbia University, New York, NY, United States; ^5^School of Medicine, Masinde Muliro University of Science and Technology, Kakamega, Kenya; ^6^Perelman School of Medicine, University of Pennsylvania, Philadelphia, PA, United States; ^7^Faculty of Medicine, Eduardo Mondlane University, Maputo, Mozambique

**Keywords:** depression, knowledge, attitude, lay health counselors, HIV/AIDS

## Abstract

**Introduction:**

Depression is the most common mental disorder among people living with HIV/AIDS and has a negative impact on HIV treatment outcomes. Training lay HIV counselors to identify and manage depression may contribute to improved patient access and adherence to treatment, and reduce stigma and discrimination among lay health workers toward both HIV and depression. The purpose of this study was to assess the current knowledge and attitudes of lay HIV counselors toward managing depression in primary care in Mozambique.

**Methods:**

We conducted a mixed-methods cross-sectional study to assess depression-related knowledge and attitudes among lay HIV counselors in 13 primary healthcare facilities in Mozambique. We used the quantitative Depression Attitude Questionnaire (DAQ) scale, followed by open-ended questions to further explore three key DAQ domains: the nature of depression, treatment preferences, and professional attitudes or reactions.

**Results:**

The sample included 107 participants (77.6% female, mean age: 32.3 years, sd = 7.4). Most (82.2%) had less than a high/technical school education. Findings suggested that some HIV counselors had knowledge of depression and described it as a cluster of psychological symptoms (e.g., deep sadness, anguish, apathy, isolation, and low self-esteem) sometimes leading to suicidal thoughts, or as a consequence of life stressors such as loss of a loved one, abuse, unemployment or physical illness, including being diagnosed with HIV infection. HIV counselors identified talking to trusted people about their problems, including family and/or counseling with a psychotherapist, as the best way for patients to deal with depression. While acknowledging challenges, counselors found working with patients with depression to be rewarding.

**Conclusion:**

Lay health counselors identified HIV and psychosocial issues as key risk factors for depression. They believed that the treatment approach should focus on social support and psychotherapy.

## Introduction

In 2019, UNAIDS estimated that 38 million people were living with HIV infection worldwide, and over 54.47% of the people with HIV and AIDS in Sub-Saharan Africa (1, 2). Mozambique is disproportionately affected by HIV and AIDS; in 2019, it had an estimated 2,183,786 people living with HIV (PWH), with 59% on antiretroviral treatment (3), and 23% who did not know their HIV status and therefore were not in treatment (2). The burden of depression among people with HIV in sub-Saharan Africa is estimated at 27% (4). The prevalence of major depressive disorder in Mozambique is 12.1% while the prevalence of depressive symptoms is 26.9% (4). Results from studies from 15 sub-Saharan countries found an estimated prevalence of depression between 6 and 59% among people living with HIV using screening tools such as the PHQ-9 (5).

Psychiatric disorders are commonly diagnosed among PWH, and depression is the most common diagnosis; some studies estimate a prevalence of over 50% (6). Depression and HIV infection have a bidirectional relationship. First, HIV infection increases the risk for depressive symptoms due to direct viral effects (HIV infects central nervous system cells), activation of the hypothalamic-pituitary-adrenal axis, increase in various inflammatory markers (TNFα, IFNγ, IL-1, and IL-6), side effects of antiretrovirals, as well as psychological stressors and vulnerabilities commonly seen in populations affected by HIV, including stigma, disability, isolation, discrimination, and poor social support (7). Second, depression has been shown to increase the progression of HIV infection, in part by affecting adherence to medications and medical care (8) as well as by its association with higher morbidity and mortality (9, 10). Depression can also be seen as a risk factor for HIV infection due to the increased likelihood of risky behaviors such as unprotected sex, multiple sex partners, and the use of intravenous drugs (7, 11). Despite the known burden and public health effects of depression on HIV infection (4), this psychiatric disorder is still underdiagnosed and undertreated in PWH (5, 12, 13).

Due to the shortage of health workers in low- and middle-income countries (LMICs) lay health counselors play a crucial role in service delivery and psychosocial support in HIV and AIDS services, mainly in primary health care (14, 15). Non-communicable disease providers in Malawi reported limited knowledge and lack of training about depression, along with inadequate resources (e.g., staff shortages, drug supply, high workload, long patient waiting times for appointments, lack of physical space) as the greatest barriers to integrating depression treatment into primary care (16). The shortage of mental health specialists in low-income countries is associated with low capacity for diagnosing and treating depression (4). To overcome this shortage, a task-shifting approach (i.e., training non-specialists to deliver mental health interventions) has been utilized in many LMICs to treat mental health problems such as depression, anxiety, and substance use in primary care and other healthcare settings (17). Mounting evidence suggests that lay health workers can be trained to deliver evidence-based treatments for depression through task-shifting when provided adequate clinical supervision (18, 19). Evidence-based interventions delivered by non-specialized health workers in primary health care settings for the treatment of depression could relieve or improve depressive symptoms and medical treatment outcomes for patients (20).

Many approaches to task shifting have been successfully tested and implemented in LMICs, indicating that through appropriate capacity building, non-mental health professionals and community health workers can assess and successfully manage mental health problems, including as part of HIV care (21, 22). As an example, depression treatment was successfully integrated into HIV care in Malawi; non-mental health care service providers were trained to screen, diagnose, and treat depression using antidepressants and psychotherapy (23–25). However, some implementation challenges arose regarding sustainability, patient retention, and providers' workloads given the limited-resource setting (23). Another study conducted in Cameroon found that primary care providers demonstrated poor knowledge and negative attitudes toward depression (26). Lay counselors often have different educational backgrounds and usually receive short-term intensive courses in counseling skills, but they do not receive any training in other mental health care topics (17, 19, 27). Skills development and technical ability are essential for effective counseling services, which goes beyond empathy and understanding of the client (17). Ongoing clinical supervision beyond the intensive training phase is a critical component of this approach (28).

Effective interventions for the treatment of depression delivered by lay health workers in low resource settings, such as group interpersonal therapy and problem-solving therapy, have demonstrated improvement in ART adherence and depressive symptoms among PWH in South Africa and Zimbabwe (29–31). Among patients with HIV, additional behavioral interventions have reduced depression relapses and improved wellness (28). However, additional studies are needed to understand the best way to implement evidence-based depression treatment (32). Gaps and challenges in integrating mental health into HIV treatment in general care health systems are evident, particularly in primary health care settings (33, 34). These gaps call for adaptation and implementation of feasible mental health interventions targeting community health workers and other stakeholders to improve the mental health of PWH (35), and have shown promising outcomes (24).

In Mozambique, lay health counselors play a key role in providing HIV counseling and testing services and psychosocial support follow-up for PWH on antiretroviral treatment (ART) (27, 36, 37). However, lay HIV health counselors are only assigned to facilities that treat a minimum of 1,000 PWH; smaller rural facilities lack support from this cadre of workers (37). The training of lay HIV counselors aims to improve overall communication, differentiate and apply diverse counseling techniques, with an emphasis on adherence counseling, and provide essential psychosocial support to PWH (36, 38). Because these HIV counselors are not trained in mental health and do not have appropriate instruments for depression screening (38, 39), referrals to mental health professionals are rare and usually occur only when there are overt changes in mood or poor adherence to ART. A task-shifting approach to mental health could minimize these challenges in HIV care if lay HIV counselors were trained to identify and manage depression (20), as they are the first contact point for PWH with health services at the primary care level (40). Notably, advocacy for mental health integration may also help to reduce HIV-associated stigma and discrimination and contribute to enhanced treatment outcomes among PWH (41). This task-shifting approach must be accompanied by adequate and continuous training, supervision, mentorship, and emotional support for lay health workers to effectively integrate mental health in HIV treatment services (14, 28, 42).

In this study, we evaluated the knowledge and attitudes of lay HIV counselors in Mozambique regarding depression to better understand the potential gaps in their practice. The secondary aim was to gather evidence that could be used to improve any future training program for lay counselors in the management of depression and other mental health related issues among PWH.

## Methods

### Participants

This study was a mixed-methods cross-sectional study investigating knowledge and attitudes among lay HIV counselors in Mozambique toward depression. Between April and November 2019, we randomly selected 13 primary health care centers from two provinces: six in Maputo Province and seven in Maputo city, and invited all HIV counselors to participate. This yielded a convenience sample of 107 providers (93.04% response rate). We considered the following eligibility criteria: currently working as a lay HIV counselor at primary care health centers (HIV, TB, Maternal and Child Health), having been a lay HIV counselor for more than 3 months, and providing written informed consent to participate in the study.

### Measures and procedures

Participants provided basic information about their clinical background as well as sociodemographic information, including age, sex, education, marital status, type of counselor, years of counseling experience, and whether they had prior training in managing mental health problems. A research assistant administered the Depression Attitudes Questionnaire (DAQ) (43). We chose the DAQ because it was relevant to the research question, available in Portuguese, and culturally adaptable to Mozambique context. The DAQ includes 20 statements with 5-point Likert Scale response options ranging from strongly agree to strongly disagree. For the analysis, we collapsed the 5-point Likert scale responses to 3 points “disagree”; “neutral” and “agree.” The 20 statements/questions are grouped into three domains. The three domains include: nature of depression (nine items: Q1, Q2, Q4, Q5, Q6, Q7, Q8, Q10 and Q11); treatment of depression (eight items: Q3, Q12, Q14, Q16, Q17, Q18, Q19, and Q20), and professional attitudes (three items: Q9, Q13 and Q15). The domain “nature of depression” corresponded to depression risk factors and clinical features (44). The “treatment of depression” domain describes the treatment orientation and confidence in types of treatment, and the “professional reaction” domain is the professional confidence and ease in managing the needs of depressed patients (45).

After completing the DAQ, each participant was asked a brief set of open-ended qualitative questions to further explore participants knowledge about diagnosis, symptoms, related risk factors, management and prevention of depression. These follow up questions were structured around the three domains of the DAQ, in order to further elucidate the participants' perspectives on these topics. For the first domain, “nature of depression,” after administering the series of structured questions from the DAQ, we asked providers to use their own words to define depression, the common symptoms, and the perceived risk factors. We also asked specifically whether they considered HIV and AIDS to be a risk factor for depression, as well as to identify barriers to mental health treatment among people with HIV. For the second domain, “treatment of depression,” after asking their agreement to various statements about treatment alternatives for depression from the DAQ, we asked participants to use their own words to describe the types of depression treatment available locally, and where the services were provided. For the third domain, “professional attitudes,” we asked counselors to describe in their own words how they help patients with depression, and if they knew of any strategies to prevent depression in people with and without HIV. The qualitative interviews were recorded and then transcribed by the research assistants. The researcher revised each transcript to verify the accuracy of the data. After transcription of the interview, the voice records were stored on a password-protected computer, only accessed by the researcher. The average duration of each interview was 25 to 30 minutes. All interviews were conducted in Portuguese.

All study procedures were reviewed and approved by the two Ethics Committees (from Federal University of São Paulo in Brazil and Eduardo Mondlane University in Mozambique).

### Mixed-methods analysis

We used an explanatory QUANT = >QUAL sequential mixed-methods approach to data collection and analysis whereby the quantitative questions were asked first, and the qualitative questions were used to further explain and expand upon each domain (46, 47). For the quantitative data, we used frequency statistics to describe the distribution of demographic characteristics, training, and clinical experience in the sample for the quantitative data. We used Stata, Version 17 for the quantitative analysis. We also calculated the internal consistency of the three DAQ subscales using Cronbach's alpha.

For the qualitative data, we used software NVIVO version 10 to file and organize verbatim transcriptions of the interviews. We utilized a directed content analytic approach wherein we began analysis examining the three sub-domains of the DAQ and then broadened our analysis to include additional pre-defined topics (47). We thereafter quantified all qualitative data by coding, thematic analysis and describing patterns that emerged in the data (48, 49). All qualitative data were coded according to predominant themes identified by FM and LFG independently. The raters worked collaboratively to develop a taxonomy of the initial themes that emerged from the data. MCG and ACS supervised the coding and re-coding at different steps of this process to verify that findings were grounded in the data. Finally, we compared the qualitative responses by domain to the quantitative findings to help interpret those observations. When there was overall agreement in the sentiment of the qualitative statements and the quantitative findings, we had more confidence that questions were being understood in the correct way. When apparent contradictions emerged between the qualitative and quantitative data, we looked to the qualitative data to try to understand if those differences were real or if another explanation–such as the misunderstanding of one or more questions–could better explain the observations.

## Results

In this sample of 107 study participants, the average age was 32.3 years (SD = 7.4), 77.6% were female, 82.2% reported their educational level as being less than middle/technical school, and 42.1% were married. About one-third of the study participants provided HIV testing and counseling, and few had prior mental health training (15.0%). On average, participants had 4 years of HIV counseling experience (range 1–15, SD = 3.5, Median = 2) (Table 1). Examination of the psychometric properties of the DAQ revealed poor internal consistency across the subscales: nature of depression (alpha = 0.393), depression treatment (alpha = 0.422), and professional attitudes (alpha = 0.338). A *post-hoc* analysis revealed that the internal consistency of the scales was slightly higher, but still relatively low, among the study participants with prior mental health training (nature of depression: alpha = 0.648; depression treatment: alpha = 0.512; professional attitudes: alpha = 0.586) (Table 2).

**Table 1:** Characteristics of the sample.

	**Full sample**,
	***n* = 107**
Age, M (SD)	32.29 (7.39)
Years of counseling experience, M (SD)	4.05 (3.46)
Nature of depression (DAQ), M (SD)	52.87 (11.11)
Professional Reaction (DAQ), M (SD)	53.19 (11.93)
Treatment preferences (DAQ), M (SD)	61.55 (11.22)
**Gender**, ***n*** **(%)**	
Female	83 (77.57)
Male	24 (22.43)
**Education level**, ***n*** **(%)**	
Basic complete	22 (20.56)
Middle/technical/pre-university incomplete or complete	66 (61.68)
Attended higher education or upper level	19 (17.76)
**Marital status**, ***n*** **(%)**	
Single	57 (53.27)
Married/civil union/living as married	45 (42.06)
Divorced, separated, or widowed	5 (4.67)
**Mental health training**, ***n*** **(%)**	
No	91 (85.05)
Yes	16 (14.95)
**Counseling type**, ***n*** **(%)**	
APSS: psychosocial counseling	29 (27.10)
Testing and counseling (ATS, ATIP, ATSC)	41 (38.32)
Specialty counselors (SMI and CCR/TB)	13 (12.15)
More than 1 type of counseling	24 (22.43)
Oher types of counseling	37 (34.58)

APSS, psychosocial and support; ATS, counseling and testing in health; ATIP, couseling and testing intiated by the provider; ATSC, Community counseling and testing, SMI, Maternal and Child health; CCR, Child at Risk Consultation and TB, tuberculosis.

**Table 2:** Psychometric characteristics.

		**Nature of depression**	**Treatment preferences**	**Professional reaction**	**Alpha if item deleted**
DAQ1	During the last 5 years, I have seen an increase in the number of patients presenting with depressive symptoms.	X			0.383
DAQ2	The majority of depression seen in general practice originates from patients' recent misfortunes (disgrace, tragedy).	X			0.361
DAQ3	Most depressive disorders seen in general practice improve without medication.		X		0.410
DAQ4	An underlying biochemical abnormality is at the basis of severe cases of depression (biochemical changes in the brain).	X			0.369
DAQ5	It is difficult to differentiate whether patients are presenting with unhappiness or a clinical depressive disorder that needs treatment.	X			0.405
DAQ6	It is possible to distinguish two main groups of depression: one psychological in origin and the other caused by biochemical mechanisms (biochemical changes in the brain).	X			0.406
DAQ7	Becoming depressed is a way that people with poor stamina deal with life difficulties.	X			0.309
DAQ8	Depressed patients are more likely to have experienced deprivation in early life than other people.	X			0.323
DAQ9	I feel comfortable in dealing with depressed patients' needs.			X	0.082
DAQ10	Depression reflects a characteristic response in patients which is not amenable to change.	X			0.395
DAQ11	Becoming depressed is a natural part of being old.	X			0.313
DAQ12	Nursing professionals working in primary care can be useful in supporting depressed patients.		X		0.437
DAQ13	Working with depressed patients is heavy going.			X	0.096
DAQ14	There is little to be offered to those depressed patients who do not respond to what GPs do.		X		0.401
DAQ15	It is rewarding to spend time looking after depressed patients.			X	0.487
DAQ16	Psychotherapy tends to be unsuccessful with depressed patients.		X		0.470
DAQ17	If depressed patients need antidepressants, they are better off with a psychiatrist than with a general practitioner.		X		0.375
DAQ18	Antidepressants usually produce a satisfactory result in the treatment of depressed patients in general practice.		X		0.369
DAQ19	Psychotherapy for depressed patients should be left to a specialist.		X		0.328
DAQ20	If psychotherapy were freely available, this would be more beneficial than antidepressants for most depressed patients.		X		0.301
	**Internal consistency**	0.3926	0.4215	0.3383	
*Secondary analysis of internal consistency by Mental health training*					
	Cronbach's alpha among people without mental health training	0.3549	0.4366	0.2913	
	Cronbach's alpha among people with mental health training	0.6484	0.5118	0.586	

There was substantial variation in the proportion of participants who agreed with certain statements regarding the nature of depression, depression treatment, and professional attitudes, which are detailed below (Table 3).

**Table 3:** Summary of quantitative findings by domain.

		**Disagree**	**Neutral**	**Agree**
	**Domain 1: Nature of depression**			
DAQ1	During the last 5 years, I have seen an increase in the number of patients presenting with depressive symptoms.	31 (28.97)	22 (20.56)	54 (50.47)
DAQ2	The majority of depression seen in general practice originates from patients' recent misfortunes (disgrace, tragedy).	18 (16.82)	14 (13.08)	75 (70.09)
DAQ4	An underlying biochemical abnormality is at the basis of severe cases of depression (biochemical changes in the brain).	37 (34.58)	31 (28.97)	39 (36.45)
DAQ5	It is difficult to differentiate whether patients are presenting with unhappiness or a clinical depressive disorder that needs treatment.	54 (50.47)	6 (5.61)	47 (43.93)
DAQ6	It is possible to distinguish two main groups of depression: one psychological in origin and the other caused by biochemical mechanisms (biochemical changes in the brain).	15 (14.02)	23 (21.50)	69 (64.49)
DAQ7	Becoming depressed is a way that people with poor stamina deal with life difficulties.	36 (33.64)	6 (5.61)	65 (60.75)
DAQ8	Depressed patients are more likely to have experienced deprivation in early life than other people.	49 (45.79)	11 (10.28)	47 (43.93)
DAQ10	Depression reflects a characteristic response in patients which is not amenable to change.	56 (52.34)	14 (13.08)	36 (33.64)
DAQ11	Becoming depressed is a natural part of being old.	71 (66.36)	8 (7.48)	28 (26.17)
	**Domain 2: Treatment preference**			
DAQ3	Most depressive disorders seen in general practice improve without medication.	32 (29.91)	11 (10.28)	64 (59.81)
DAQ12	Nursing professionals working in primary care can be useful in supporting depressed patients.	13 (12.15)	7 (6.54)	86 (80.37)
DAQ14	There is little to be offered to those depressed patients who do not respond to what GPs do.	68 (63.55)	15 (14.02)	24 (22.43)
DAQ16	Psychotherapy tends to be unsuccessful with depressed patients.	78 (72.90)	12 (11.21)	17 (15.89)
DAQ17	If depressed patients need antidepressants, they are better off with a psychiatrist than with a general practitioner.	7 (6.54)	5 (4.67)	95 (88.79)
DAQ18	Antidepressants usually produce a satisfactory result in the treatment of depressed patients in general practice.	12 (11.21)	21 (19.63)	74 (69.16)
DAQ19	Psychotherapy for depressed patients should be left to a specialist.	19 (17.76)	7 (6.54)	81 (75.70)
DAQ20	If psychotherapy were freely available, this would be more beneficial than antidepressants for most depressed patients.	11 (10.28)	10 (9.35)	85 (79.44)
	**Domain 3: Professional reaction**			
DAQ9	I feel comfortable in dealing with depressed patients' needs.	26 (24.30)	15 (14.02)	66 (61.68)
DAQ13	Working with depressed patients is heavy going.	94 (87.85)	5 (4.67)	8 (7.48)
DAQ15	It is rewarding to spend time looking after depressed patients.	6 (5.61)	10 (9.35)	91 (85.05)

Domain 1: Nature of depression; Domain 2: Treatment preference and Domain 3: Professional reaction.

### Domain 1: Nature of depression

Almost all (98.1%) participants reported that they had heard of depression, but fewer than half of the participants reported personally knowing someone with depression. The majority of the counselors reported having learned about depression through their professional practice, while a few learned about it through in-service training and academia or had found information about depression through reading books. On the DAQ scale, a majority (64.49%, *n* = 71) disagreed that “becoming depressed is a natural part of being old” (question 11) and 52.34% (*n* = 56) disagreed that “depression reflects a characteristic response in patients which is not amenable to change” (question 10). Almost half of participants 50.47% (*n* = 54) agreed that during the last 5 years, [they] have observed an increase in the number of patients presenting with depressive symptoms (question 1) and most (70.09%, *n* = 75) agreed that “the majority of depression seen in general practice originates from patients' recent misfortunes (disgrace, tragedy)” (question 2). However, many counselors 60.75% (*n* = 65) revealed some stigmatizing attitudes by agreeing that becoming depressed is a way that “fragile” people deal with life's difficulties (question 7) (Table 3).

For other statements relating to the nature of depression, including biochemical and biological origins, there was limited agreement (34.58%, *n* = 37 disagreed; 28.97%, *n* = 31 were neutral; and 36.45%, *n* = 39 agreed) among participants (question 4); 60.75% (*n* = 69) considered that it was possible to distinguish different presentations of depression (psychological, biochemical, or related to adversity); and almost half 45.79% (*n* = 49) of the participants believed that patients with depression probably experienced more childhood deprivation than other people (question 8) (Table 3). When asked about risk factors for depression, only a few participants considered heredity as a biological risk factor, whereas most cited psychosocial factors such as marital, social, and financial problems; unemployment; and trauma, sexual, physical, or verbal violence happening in childhood and adulthood.

Subsequent qualitative inquiry revealed a more nuanced conceptualization of depression, characterized in two ways. The first description included one or more of the following words: a psychological or psychiatric illness that is characterized by a feeling of deep sadness, anguish, apathy, altered mood, isolation, low self-esteem, inactivity, guilt, and sometimes suicidal thoughts or ideation.

“*Depression is a psychiatric illness characterized by a feeling of deep sadness, apathy, mood swings, inactivity, and sometimes suicidal thoughts or ideation…”* Male HC, 46 years old

The second type was depicted as more of an emotional reaction to unexpected stressful situations (e.g., results of an HIV test) and difficulties in dealing with problems.

“*(depression) is a disease that affects people with difficulties in overcoming certain problems in life; when it is not treated, it can become chronic and lead to suicide…”* Female HC, 36 years old

Participants described depression in five main ways: (1) isolation (feeling alone, social isolation, being isolated, abandoned, not wanting to be with others, withdrawing, bashful, staying in your corner, hiding); (2) sadness (deep sadness, feeling depressed, distraught, disappointed, dysphoric mood; negative mood and behavior); (3) apathy (weak, haggard, passive); (4) restlessness (being out of your mind, becoming stressed, upset); and (5) suicidal ideation (loses desire to live, loses interest in living, thinking about wanting to die, wanting to take one's life) (Table 4).

**Table 4:** Symptoms of depression.

**Symptoms of depression**	***n***	**%**
Isolation (isolation, feeling alone, social isolation, being isolated, abandoned, not wanting to be with others, withdrawing, bashful, staying in your corner, hiding)	32	18.9
Sadness (stay / sad, sad mood, deep / advanced sadness)	29	17.2
Depressed, weak, haggard, distraught, disappointed, state of mood and negative behavior, apathy, passive	16	9.5
Dysphoric mood, restlessness, be out of your mind, become stressed, upset	11	6.5
Suicide ideation (loses desire to live, lose interest in living, thinking want to die, want to take one's life)	11	6.5
Lack pleasure (loses will to do things you love, unwilling to do anything, to feel inhibited in things, do not accept the things you love, stop doing activities that previously did, inactivity)	10	5.9
Desperate, demoralized, inferior, weakened, devalued	9	5.3
Difficulty talking (not want to talk to nobody is not want to share, do not want to talk, afraid to open up, unwilling to count the problems)	9	5.3
Lack of interest in many aspects of life, nothing has importance, feel guilty, not being satisfied with your life	9	5.3
Thinking that life stopped, unable to deal with problems, do not accept the reality	7	4.1
Low self esteem	6	3.6
Neglected, lack of hygiene care, no bathing	6	3.6
Physical and psychological discomfort, confusion, inactivity, thinking about pain and hurt	5	3
Crying	4	2.4
Lack appetite, do not eat, lose weight	3	1.8
Difficulty to withhold certain information and difficult to face situations	2	1.2
Total	169	100.0

When asked about risk factors for depression, participants mentioned low self-esteem; lack of resilience; heredity (genetic predisposition); isolation; marital, social, and financial problems; unemployment; trauma (verbal, physical, sexual, or psychological violence); alcohol and drug abuse; and/or death or loss of loved one. Nearly all believed HIV was a risk factor for depression due to learning of the HIV diagnosis as a chronic disease requiring medication for life, as well as introducing problems of discrimination, stigmatization, and prejudice (Table 5).

**Table 5:** Depression factors.

	***n***	**%**
Lack of resilience, isolation, marital, social, financial problems, unemployment.	39	36.45
Diagnostic disclosure of HIV and chronic disease	21	19.63
Death or loss of a loved one	17	15.89
Trauma, violence verbal, physical, sexual, psychological and abuse of alcohol and drugs	15	14.02
Low self-esteem, discrimination, stigmatization and prejudice	13	12.15
No reply	2	1.87

The four of the most commonly cited risk factors for depression were marital, social, financial, and unemployment problems followed by learning the diagnosis of chronic conditions such as HIV; death or loss of loved ones; and traumatic experiences during childhood and adulthood (verbal, physical, psychological, domestic and sexual abuse). Other risk factors mentioned were discrimination, stigmatization and prejudice, solitude and isolation, anxiety and worry, difficulty expressing oneself, unhealthy lifestyle (addiction, alcohol and substance abuse), low self-esteem, lack of resilience, expression difficulty, being postpartum, and heredity (Table 5).

“*… Loss of a loved one, consumption of psychoactive substances, unresolved grief, job loss, unemployment and chronic illnesses …”* Female HC, 37 years old

“*… Abuses and mistreatment in childhood without opening up to someone can cause trauma in adult life; cost of living and wars that can lead to future depression…”* Female HC. 37 years

“*… Lack of employment, lack of family support, discrimination and grief …”* Female HC, 41 years old

#### HIV as a risk factor

Almost all participants believed that HIV was a risk factor for depression. The association between HIV and depression was explained through difficulty accepting the test result; fear of disclosing the diagnosis to a partner and/or family member; fear of discrimination; hopelessness; difficulty facing HIV and AIDS as a chronic disease without cure requiring treatment for life. According to the health counselors, the main problems described by HIV positive patients that could increase the risk of depression were lack of family support, discrimination, and lack of support and conflict with a partner; not speaking or speaking little; not knowing what depression is; difficulty/fear in disclosure to the partner and acceptance of the diagnosis; taking medication and its side effects; poverty, hopelessness, financial/transportation/food difficulties; and poor treatment (Table 6).

“*… Generally speaking, it can (cause depression). Those people who do not want to accept their status, think that HIV is the end of life and the person alone begins to feel discriminated against without others knowing what their status is …”* Female HC, 33 years old

“*… Yes, for example, when the person is tested and the result is positive (for HIV), he goes into depression because he thinks it is the end of his life and he is afraid to tell his family.…”* Female HC, 34 years

“*… Currently, since the care is integrated and people are informed about HIV, (depressive symptoms) do not develop. Years ago, people could develop depression because they thought it was the end of life …”* Male HC, 24 years old

“*… I think (HIV can cause depression), first because it is a disease that is not curable, and second, because the person with HIV is vulnerable to being discriminated against, the long-time treatment, and at some point, the side effects of the treatment …”* Male HC, 41 years old.

**Table 6:** HIV/AIDS associated with depression.

	***n***	**%**
People find it difficult to accept their serostatus in the initial phase, because they know that HIV is a chronic disease, with no cure and because they comply with treatment for life, a factor that can lead to depression	73	68.2
Sociocultural issues, habits, beliefs and values associated with HIV culminate in discrimination, stigma and lack of support by the partner and family as well as the community	27	25.2
I believe that HIV is not a risk factor for depression	4	3.7
If the form of revelation by the health provider to the patient is not preceded by psychological preparation, this can lead to depression	3	2.8

#### Problems reported by PWH

Providers also talked about the main problems described to them by patients, which included the lack of social and financial support, lack of partner and family support, discrimination, stigma, and interpersonal conflict. Other patient concerns reported by the counselors were uncertainty, the fear of revealing their diagnosis to their partners, and the fear of medication's side effects (Table 7).

“*… There are people who talk. Sometimes they talk about lack of money for transport and food, abuse in the family and even in the hospital, they face some shame…”* Male HC, 60 years old

“*… They even talk when we do not have time to talk to them. Many of them do not open up. The problems are poverty, the illness itself and frequent losses in the family …”* Female HC, 28 years old

“… *Yes, they do (talk about depression). (They) often question whether they still will be the same individuals and are concerned too often with medication and what family or friends think of it ..*.” Female HC, 33 years

“*… The lack of acceptance of the diagnosis and the fact that he is not debilitated makes him not accept his status …”* Male HC, 23 years old

“*…Some talk (about their problems), that they feel alone, that they don't have enough money (for transportation), stigma, lack of family support, non-acceptance of the (HIV) positive diagnosis and fear of going to the health facility…”* Male HC, 26 years old.

**Table 7:** Lay counselors' description of barriers reported by patients.

**Main (barriers) reported by patients**	***n***	**%**
Lack of partner and family support, discrimination, stigma and conflict	34	31.8
Do not speak or speak little and some do not know what is depression	30	28
Difficulty/fear in disclosing the partner and accepting the diagnosis, taking medication, side effects and poor care	29	27.1
Socio-economic factors such as poverty, unemployment, financial difficulties, transport and food	14	13.1

#### Depression prevention

The most cited preventive mechanisms for depression were communication (conversation/dialogue) with family members or trusted people about problems and concerns; seeking support from friends; counseling and follow-up with a psychologist; and self-esteem, socialization, avoiding stress, stigma, violence, and other life situations. The other forms of prevention were disseminating information and lectures on depression through social media (TV, radio), relaxation, and sports to cope with problems (Table 8).

“*… Talking more to people, someone trustworthy exposing the problems. Having the habit of looking for a psychologist to express concerns…”* Female HC, 29 years old

“*… Talk to trusted and experienced people in case of any discomfort or difficulty, seek support at the level of health units for better clarification…”* Female HC, 37 years old

“… *More community involvement and events, as an alternative for dissemination* …” Male HC, 26 years old

“… *First, there must be dialogue in a family, health providers must try to listen or listen to the patient and help him* …” Female HC, 36 years old

“… *I believe that conversation and counseling help to distract the mind so that the person does not always think about being HIV positive* …” Female HC, 38 years old

**Table 8:** Depression prevention mechanisms reported by lay health counselors.

**Depression prevention mechanism**	***n***	**%**
Communication (conversation/dialogue) with family or trusted people about your problems and concerns.	29	27.1
Seek support from friends, counseling and follow-up at the psychologist	21	19.6
Self-esteem, socialization, avoiding and dealing with stress, stigma, violence and other life situations.	19	17.8
Dissemination of information and lectures on depression (TV, radio, community).	17	15.9
Does not know or considers that there is no prevention.	11	10.3
Relaxation, sports and facing problems.	10	9.3

### Domain 2: Treatment of depression

Most lay HIV counselors supported psychotherapy and medication to treat depression but most believed mental health specialists should provide it. The majority 75.70% (*n* = 81) agreed with the statement “psychotherapy in patients with depression should be left to the specialist” (question 19) and 59.81% (*n* = 64) agreed with the statements “most depressive disorders seen in general practice improve without medication” (question 3) and 80.37% (*n* = 86) believed that “nursing professionals working in primary care can be useful in supporting depressed patients” (question 12). Many 63.55% (*n* = 68) did not endorse the statement “there is very little to offer patients with depression who do not get better with the treatment proposed by the general practitioner” (question 14); however 72.90% (*n* = 78) disagreed that “psychotherapy in patients with depression does not usually give good results” (question 16), and 79.44 (*n* = 85) agreed that “if psychotherapy were freely available, it would have more benefits than antidepressants for most patients with depression” (question 20) (Table 3). When asked qualitatively, the counselors reported that they believed it was possible to treat depression, and this treatment could be through a psychologist, psychiatric technician or specialist for medications and the other option was counseling, psychosocial support and/or through support groups (family and community) (Table 9).

“*… I think it is possible (to treat depression), there are several forms of treatment, through counseling, empathy, medication, lectures and socializing with people who have already been through the same situation.…”* Male HC, 24 years old

“*… It is (treatable), depending on the type, there are cases in which the treatment can be through physical exercises, some relaxation techniques. In other cases, specific treatment with antidepressants indicated by the specialist is necessary.…”* Male HC, 41 years old

“*… I think so, doing some sessions with psychologists or psychiatrists…”* Female HC. 30 years

“*Some people have chronic depression, which is difficult to treat but still treatable. It is talking to the person or meeting with a psychologist …”* Female HC, 36 years old

**Table 9:** Types of treatment for depression.

**Forms/types of treatment for depression**	***n***	**%**
Counseling, psychological and drug treatment	77	72.0
Counseling and family and community support sessions	29	27.1
None	1	0.9

For the treatment of depression, participants suggested patients could seek psychological and psychiatric consultation with a doctor or trained counselor at health facilities (health center, hospital, pharmacy, clinic) or home, with family and/or friends. The participants said that at health facilities, treatment for depression could be found at the following services: Health Counseling and Testing, Psychosocial Support, and Young and Adolescent Friendly Services (Table 10).

**Table 10:** Treatment setting.

**Where can you find treatment for depression**	***n***	**%**
Psychology and psychiatry consultation, physician, trained counselor	34	31.8
The health facility (center health, hospital, pharmacy, clinic).	32	29.9
In hospitals where there are psychologists.	17	15.9
At the health services, at home (talking to a health provider or a family member or friends)	15	14

### Domain 3: Professional attitudes

Most participants 85.05% (*n* = 91) agreed that it was rewarding to spend time caring for depressed patients (question 15) and 87.85% (*n* = 94) disagreed that working with depressed patients is “heavy going” (question 13). Most lay HIV counselors 61.68% (*n* = 66) reported feeling comfortable dealing with depressed patients' needs (question 9). Participants suggested the strategies for helping patients presenting with depressive symptoms were: to refer to a psychologist, psychiatric technician, or specialist; conversation, welcoming, and counseling; and demonstrating empathy and actively listening to the patients' difficulties (Table 11).

**Table 11:** Lay health counselors attitude toward depression.

**Health counselors attitude toward patient with depression**	***n***	**%**
Refer to psychologist, psychiatry technician or specialist.	57	53.3
Conversation, Reception and Advice.	32	29.9
Create empathy and active listening to the patient's difficulties.	15	14
Communication (conversation/dialogue) with family or trusted people about your problems and concerns.	3	2.7

## Discussion

This is, to our knowledge, the first study exploring knowledge and attitudes about mental health among lay HIV counselors in Mozambique. The key findings in our study are: first, the counselors demonstrated limited knowledge regarding the biological risk factors for depression (such as genetic and biochemical changes in the brain) and considerable knowledge about depression symptoms, psychosocial risk factors and management. Few had received specific or formal training in mental health and thus much of their knowledge about the presentation and management of depression was acquired through self-learning, books, or information from the media. Participants' conception of depression was based on their experiences in dealing with HIV positive patients in their daily routine, with considerable social and financial hardship. Most providers believed healthy lifestyles, family and community support, counseling, psychotherapy, medication treatment with mental health specialists could help to prevent and treat depression.

In contrast with other settings, non-mental health specialists and family practitioners in Saudi Arabia displayed high levels of knowledge and positive attitudes regarding depression and anxiety (50); this was attributed to their additional training in the management of mental disorders. When the R-DAQ was used among non-psychiatric physicians in Saudi Arabia, they showed optimism and confidence in managing depression, a positive attitude toward depression, but discriminatory and stigmatizing explanations for causes of depression–such as lack of willpower, poor stamina, part of the aging process (51). Furthermore, participation in any training related to mental health during the previous 5 years improved attitudes by 17% compared to other professionals (45). Moreover, despite their lack of mental health training, lay health counselors did not report religious or supernatural explanations for depression (such as witchcraft, evil spirits, divine punishment), which were reported by physicians not specialized in psychiatry in Pakistan (52) and by church volunteers in Nigeria (53). The participants demonstrated weak knowledge regarding the nature of depression. Only 52% disagreed that “depression reflects a characteristic response in patients which is not amenable to change” (DAQ 10) and 60% considered depression to be a way that “fragile people deal with life's difficulties” (DAQ 7). The former observation is similar to what was found in a study among general practitioners in Brazil (54). Similarly, consideration that depression is how fragile people deal with life's difficulties was also observed among nurses in Spain before receiving training on mental health (44). These findings suggest that more targeted training on depression is needed, with active components of stigma reduction to address negative stereotypes and false conceptions.

In our study, most participants had heard of depression, although most did not personally know people with depression. Most believed that depression was associated with HIV and AIDS. Providers also recognized the relationship between depression and other chronic conditions as contributing to low self-care with negative impact to patients' health (55). HIV and AIDS leads to depression through stigma and self-blame (56) or because of the challenge of accepting the diagnosis and the chronic demands involved in taking medications. Lack of income associated with unemployment and other difficulties associated with subsistence living, older age, distance to facility, stigma, and concerns about diagnostic disclosure and side-effects from ART (5, 57, 58) were also reported in our study as mental health stressors. Fear of disclosing one's HIV status to their partners can reduce the likelihood that people will adopt protective measures (such as condom use), which can increase the risk of transmission to others (59–62). Futhermore, HIV disclosure can have negative consequences such as stigma and discrimination, divorce and partner violence (63, 64) which are risk factors for depression (65). Disclosure of HIV serostatus has also been related to optimal adherence and freedom to use ART (58) as well as increased emotional and financial support (63, 66).

Our sample considered counseling, psychological and drug treatment and effective treatment strategies. The use of antidepressants was less frequently mentioned, which may be related to a false belief that treating depression with antidepressants may have a negative impact on HIV treatment adherence and outcomes (67). This is consistent with the fact that most counselors felt that psychologists and psychiatrists should manage depression, and that the HIV counselors lacked the skills to manage it. As seen in our study, active and empathic listening of patients' concerns, distress, and questions about treatment were seen as ways to improve engagement in treatment. Similar findings have been reported in studies conducted among other types of healthcare professionals, such as pharmacists (68).

The preventive measures and treatments reported by lay health counselors are in line with those recommended for populations outside of Mozambique, which include physical and recreational activity, social interactions, relaxation techniques, and seeking counseling either by professionals (licensed psychotherapists, psychiatrists, family medicine physicians, pharmacists) or by family members, friends, and acquaintances (69). This is consistent with the answers provided in the DAQ which suggested counselors believed that psychotherapy is more beneficial than antidepressants for the treatment of depression.

Training health providers to manage mental illness, mainly depression, is essential in resource-limited settings, and such training has been shown to improve knowledge and competence, as well as the delivery of effective mental health services (26, 45, 50). There was general agreement, not only among the lay health counselors but other non-mental health providers, that a psychiatrist or psychologist would better manage depression, not only because of the lack of integration of mental health services in primary care settings but also because of self-reported challenges in the identification of depressive symptoms by primary care providers (26, 45, 54, 70–72); there is ample evidence demonstrating that both lay health counselors and other non-mental health providers can be trained to effectively identify depression (73, 74). Mental health training may improve depression-related knowledge, attitudes, and practices in this group (45, 75). Our study suggests that training lay health counselors has the potential to improve their knowledge and attitudes toward depression in PWH and also care and outcomes as part of mental health in HIV services within primary healthcare setting.

## Study limitations

The data for this study were collected in primary care health centers at the level of primary care located in Maputo city and Maputo province, so they may not be generalized to the rest of the country. The instrument for this study may be subject to information bias due to the poor mental health literacy of the participants. That said, one strength of our study–in light of the low internal consistency of the DAQ instrument–was the qualitative component. After each set of quantitative “agreement statements” by domain, we encouraged participants to use their own words to describe the same concepts. This extra step proved invaluable to us in order to be able to interpret some of the findings that were apparently in contrast. The small sample size for the quantitative survey limits the generalizability of its findings; however, having collected a small amount of qualitative data from such a large sample was a strength in this study. Other studies have also shown weak internal consistency of the DAQ in general and its subscales (45, 76). Since the qualitative interviews were conducted after administering the DAQ, the degree to which the answers may have been influenced by the survey questions is unclear. For this reason, it is reasonable to suggest adjustments that include additional items tailored to the professionals being assessed (71).

The DAQ is a tool primarily used to determine attitudes toward depression among medical doctors and other health professionals, but one of its limitations is low internal consistency (70). Studies provided insufficient information regarding psychometric properties and did not include internal consistency (45, 76). In the first Portuguese version study of the DAQ, the factorial analysis was omitted due to the small sample size for each item (54).

## Conclusions and implications

This study describes the knowledge and attitudes of lay HIV counselors regarding depression associated with their daily practice/routine, especially those already providing psychosocial support. Training lay counselors in mental health is associated with better knowledge, attitudes, and practices in LMICs (75). Additional research will be needed to understand sustainable ways of incorporating evidence-based interventions and community-based participatory methods in order to reduce mental health disparities (73). Training and supervising lay health workers to deliver effective counseling interventions (39) can address depression among PWH in LMIC. Since lay counselors are the first point of contact for PWH in primary care, and given the shortage of mental health professionals, the training of HIV counselors in the identification and management of depression could enhance access to and improvements in care. Depression among PWH is associated with poor social connections, lower help-seeking, and adverse health behaviors (77). Health counselors have the potential to improve patient care in HIV clinics. By improving their competency to screen for and manage depression, they can potentially mitigate the negative impact of depression on adherence to ART and, hopefully, improve the prognosis of HIV infection.

Understanding medical practitioners' attitudes can guide and assess the needs for training at the primary healthcare level. This is also true for lay HIV counselors, who organize service delivery specifically for PWH and manage treatment outcomes (54, 78). Of note, most of our participants accurately linked the conditions of depression and HIV/AIDS.

Basic brief training positively impacts non-mental health professionals' knowledge and attitudes toward depression and impacts patients' education and care, especially for those with chronic disease and/or involved in long-term treatment (79, 80). Investing in the training of lay health counselors can significantly impact patient care for PWH and people with other chronic illnesses where depressive symptoms may affect patients' quality of life, adherence, and prognosis.

## Data availability statement

The raw data supporting the conclusions of this article will be made available by the authors, without undue reservation.

## Ethics statement

The studies involving human participants were reviewed and approved by Ethics Committees (from Federal University of São Paulo in Brazil and Eduardo Mondlane University in Mozambique). The patients/participants provided their written informed consent to participate in this study.

## Author contributions

FM, MG, LP, and AS drafted the manuscript. MG did statistical analysis. AS did qualitative analysis. MG, CD, FC, MS, ES, DK, MO, and MW reviewed the manuscript. FM and MM designed the protocol. All authors agreed to the final version of the manuscript.

## Funding

This study was supported by NIMH grants U19MH113203 and T32MH096724; and Fogarty International Center grant D43TW009675.

## Conflict of interest

Author MO receives royalties from the Research Foundation for Mental Hygiene for the Columbia Suicide Severity Rating Scale's commercial use and owns shares in Mantra, Inc. She serves as an advisor to Alkermes and Fundacion Jimenez Diaz (Madrid). Her family owns stock in Bristol Myers Squibb. The remaining authors declare that the research was conducted in the absence of any commercial or financial relationships that could be construed as a potential conflict of interest.

## Publisher's note

All claims expressed in this article are solely those of the authors and do not necessarily represent those of their affiliated organizations, or those of the publisher, the editors and the reviewers. Any product that may be evaluated in this article, or claim that may be made by its manufacturer, is not guaranteed or endorsed by the publisher.

## Abbreviations

AIDS: Acquired Immunodeficiency Syndrome
ART: Antiretroviral Treatment
ARV: Antiretroviral
ART: Highly Active Antiretroviral Therapy
DAQ: Depression Attitude Questionnaire
HIV/AIDS: Human Immunodeficiency Virus/ Acquired Immunodeficiency Syndrome
IL: Interleukin (IL-1IL-6)
IFNγ: Interferon gama
LMIC: Low and Middle Income Country
PWH: People Living With Human Immunodeficiency Virus/ Acquired Immunodeficiency Syndrome
PHQ-9: Patient Health Questionnaire 9
TB: Tuberculosis
TNFα: Tumor Necrosis Factor alfa.
